# Acceptability and Usability of the Family Gene Toolkit for Swiss and Korean Families Harboring *BRCA1/BRAC2* Pathogenic Variants: A Web-Based Platform for Cascade Genetic Testing

**DOI:** 10.3390/cancers15184485

**Published:** 2023-09-09

**Authors:** Vasiliki Baroutsou, Vu Duong, Alice Signorini, Ramon Saccilotto, Florina M. Ciorba, Nicole Bürki, Maria Caiata-Zufferey, Jai Min Ryu, Sung-Won Kim, Myong Cheol Lim, Christian Monnerat, Ursina Zürrer-Härdi, Jisun Kim, Karl Heinimann, Rossella Graffeo, Ji Soo Park, Manuela Rabaglio, Pierre Olivier Chappuis, Sue Kim, Maria C. Katapodi, on behalf of the CASCADE and K-CASCADE Consortia

**Affiliations:** 1 Department of Clinical Research, Faculty of Medicine, University of Basel, 4055 Basel, Switzerland; vasiliki.baroutsou@unibas.ch (V.B.); vu.duong@usb.ch (V.D.); alice.signorini@stud.unibas.ch (A.S.); ramon.saccilotto@usb.ch (R.S.); 2 Department of Mathematics and Computer Science, University of Basel, 4051 Basel, Switzerland; florina.ciorba@unibas.ch; 3 Women’s Clinic, University Hospital Basel, 4031 Basel, Switzerland; n.buerki@hin.ch; 4 Department of Business Economics, Health and Social Care, University of Applied Sciences and Arts of Southern Switzerland, 6928 Manno, Switzerland; maria.caiata@supsi.ch; 5 Division of Breast Surgery, Department of Surgery, Samsung Medical Center, Sungkyunkwan University School of Medicine, Seoul 06351, Republic of Korea; jaimin.ryu@samsung.com; 6 Department of Breast Surgery, Breast Care Center, Dairim St Mary’s Hospital, Seoul 07442, Republic of Korea; brcakorea@gmail.com; 7 Division of Tumor Immunology, Center for Gynecologic Cancer Research Institute and Hospital, National Cancer Center, Goyang 10408, Republic of Korea; mclim@ncc.re.kr; 8 Department of Medical Oncology, Hospital of Jura, 2800 Delemont, Switzerland; christian.monnerat@h-ju.ch; 9 Department of Medical Oncology, Cantonal Hospital Winterthur, 8400 Winterthur, Switzerland; ursina.zuerrer@ksw.ch; 10 Department of Surgery, Asan Medical Center, University of Ulsan College of Medicine, Seoul 05505, Republic of Korea; jisunkim@amc.seoul.kr; 11 Institute for Medical Genetics and Pathology, University Hospital Basel, 4031 Basel, Switzerland; karl.heinimann@usb.ch; 12 Research Group Human Genomics, Department of Biomedicine, University of Basel, 4031 Basel, Switzerland; 13 Oncology Institute of Southern Switzerland, Ente Ospedaliero Cantonale (EOC), 6500 Bellinzona, Switzerland; rossella.graffeogalbiati@eoc.ch; 14 Hereditary Cancer Clinic, Cancer Prevention Center, Yonsei Cancer Center, Division of Medical Oncology, Department of Internal Medicine, Yonsei University College of Medicine, Seoul 03772, Republic of Korea; pmjisu@yuhs.ac; 15 Department of Medical Oncology, Inselspital, Bern University Hospital, 3010 Bern, Switzerland; manuela.rabaglio@insel.ch; 16 Unit of Oncogenetics, Division of Precision Oncology, University Hospitals of Geneva, 1205 Geneva, Switzerland; pierre.chappuis@hcuge.ch; 17 Division of Genetic Medicine, University Hospitals of Geneva, 1205 Geneva, Switzerland; 18 College of Nursing, Yonsei University, Seoul 03722, Republic of Korea; suekim@yuhs.ac

**Keywords:** active coping, decisional support, family communication, genetic counseling, HBOC, psychoeducational intervention, Tier 1 genetic condition

## Abstract

**Simple Summary:**

The study adapted an existing Web-based intervention, the Family Gene Toolkit, for Swiss and Korean families that harbor the genetic changes associated with hereditary breast and ovarian cancer syndrome. The Family Gene Toolkit encourages family communication of genetic testing results and cascade genetic testing among at-risk relatives. Feedback from 68 women with genetic changes and 31 clinicians informed the culturally sensitive adaptation of the content. The Information Technology team developed the web application that will host the intervention. Finally, a new sample of 18 women from families with hereditary breast and ovarian cancer reviewed and evaluated the adapted content and the functions of the web application. Findings support that overall, the adapted Family Gene Toolkit is well-designed, has useful information for these families, and provides interactive content and illustrative stories. The research team will test if it can increase rates of cascade testing among at-risk relatives in a subsequent randomized trial.

**Abstract:**

The study adapted the Family Gene Toolkit and developed a customized web application for Swiss and Korean families harboring *BRCA1* or *BRCA2* pathogenic variants to support family communication of genetic testing results and promote cascade genetic testing among at-risk relatives. In the first step, narrative data from 68 women with *BRCA1*/*BRCA2* pathogenic variants and clinician feedback informed a culturally sensitive adaptation of the content consistent with current risk management guidelines. In the second step, the Information Technology team developed the functions and the interface of the web application that will host the intervention. In the third step, a new sample of 18 women from families harboring *BRCA1*/*BRCA2* pathogenic variants tested the acceptability and usability of the intervention using “think-aloud” interviews and a questionnaire. Participants expressed high levels of satisfaction with the intervention. They provided positive feedback for the information regarding active coping, strategies to enhance family communication, interactive elements, and illustrative stories. They reported that the information was useful and the web application was easy to navigate. Findings suggest that the Family Gene Toolkit is well-designed and can increase rates of cascade testing among at-risk relatives. Its efficacy will be tested in a subsequent randomized trial.

## 1. Introduction

Clinical practice guidelines recommend testing individuals diagnosed with cancer to identify carriers of germline pathogenic variants [1]. Upon identifying a germline pathogenic variant, offering cascade genetic testing to cancer-free relatives promotes primary and secondary cancer prevention. Cascade testing for Hereditary Breast and Ovarian Cancer syndrome (HBOC) has been described by the Centers for Disease Control and Prevention (CDC) as a Tier 1 genetic application [2]. HBOC is diagnosed in about 5–10% and about 20% of all breast and ovarian cancer cases, respectively, with some estimates being higher for selected patients and families [3]. HBOC is also implicated in prostate, pancreatic, and skin cancer, as well as in other malignancies [4].

Despite calls to action for cascade testing of biological relatives of HBOC cases, there are barriers to its implementation. Privacy laws worldwide prohibit healthcare providers from reaching at-risk relatives without the explicit consent of the tested individual [5]. The responsibility to share genetic test results lies exclusively with the individual carrying the pathogenic variant, who may simultaneously be struggling with a cancer diagnosis [6,7,8]. This communication strategy has significant limitations in ensuring contact with at-risk relatives and the transmission of accurate information [9,10], leaving approximately 50% of them unaware of their potential cancer risk [11]. This created the challenge of reaching relatives who are not in contact with the healthcare system through family networks [12,13,14,15]. Genetic specialists responded by writing family letters that can be distributed by the tested individual or sent directly to at-risk relatives. However, family letters have been implemented inconsistently due to increased clinician burden, and studies have shown mixed results [16,17].

Interventions supporting individuals with HBOC-associated variants during the disclosure of genetic test results have the potential to reduce their psychological distress. Additionally, such interventions can serve to provide relatives with accurate and dependable information about cascade testing. They also need to minimize the efforts of genetic specialists while abiding by existing legal frameworks regarding the privacy and confidentiality of genomic information. Given the explosion of health communication technologies [18], novel approaches are needed. Technology-enabled health communication is equally effective in disseminating accurate information, is cost-effective, and can increase access to services [19,20,21]. Leveraging digital health communication is also consistent with consumer behavior since about 20% of families use social media to share genetic testing results [22], and more than 80% of individuals use the World Wide Web to acquire health-related information [23,24,25]. However, there are only a handful of trials regarding family communication of genetic testing results and/or cascade genetic testing [26]. Few studies involving digital communication technologies, such as chatbots or other digital media, describe pilot testing in non-randomized trials and/or without comparisons to a control group [27,28,29,30,31,32,33].

We developed a web-based family intervention, the Family Gene Toolkit, to encourage disclosure of genetic testing results from individuals with *BRCA1* or *BRCA2* pathogenic variants and support cascade testing among at-risk relatives [34]. The prototype was based on the theory of stress and coping [35] and adapted to the needs of HBOC families, i.e., individual and family adaptation to genetic illness [36,37] and decision-making [38]. The prototype addressed genetic predisposition to cancer and the accuracy of genetic testing. A decisional support tool included patient testimonials about accepting or refusing genetic testing based on the International Patient Decision Aids Standards criteria [39]. The prototype was delivered by a certified genetic counselor and a master’s prepared oncology nurse using two live webinars (PowerPoint presentations with live audio) and one brief follow-up phone call [34]. Live webinars enabled real-time interaction among family members and expert clinicians and lasted approximately 60 min. The first webinar was facilitated by the genetic counselor and provided information about cancer genetics, counseling, and testing. The second webinar was offered a week later by the oncology nurse and provided information on active coping strategies and the effective communication of genetic testing results. Two weeks following the second webinar, each participant received a 15-min phone call from the genetic counselor and the nurse to address individual concerns.

The Family Gene Toolkit prototype was tested with U.S.-based participants. Acceptability and usability were tested with focus groups, while feasibility and efficacy were tested in a pilot study using a randomly assigned wait-listed control group. Results provided proof of concept for the high acceptability, usefulness, participant satisfaction, and efficacy of the intervention [34]. However, findings also highlighted issues that would impede the upscale of implementation. Scheduling live webinars to accommodate the lifestyle and different time zones of family members and clinicians was interfering with the success of the approach. The involvement of two master’s prepared clinicians made for an expensive intervention and raised questions about its cost-effectiveness. There was also a lack of consensus about the optimal time frame for intervening, indicating variability in preferences due to competing priorities, e.g., cancer treatment or relatives’ life trajectories. Live webinars precluded the possibility of tailoring the timing of delivering the intervention to individual circumstances and preferences.

The purpose of this study was to describe the adaptation of the Family Gene Toolkit prototype for upscaling its implementation in clinical practice. Adapting and expanding an existing prototype, rather than developing a new intervention, takes advantage of previous valid experiences without duplicating efforts. The adapted Family Gene Toolkit also addresses the changing informational requirements of international audiences, specifically Swiss and Korean families. Although Swiss and Korean populations are ancestrally different, the prevalence of *BRCA* pathogenic variants is comparable between the two countries, along with a growing interest and concern about HBOC in Korea [40,41,42]. The culturally sensitive adaptation of digital health communication interventions is extremely timely and relevant, given the expansion of genetic technologies, the falling costs of testing, and the increased pressure for integrating genetic knowledge into practice.

## 2. Materials and Methods

**Figure 1:**
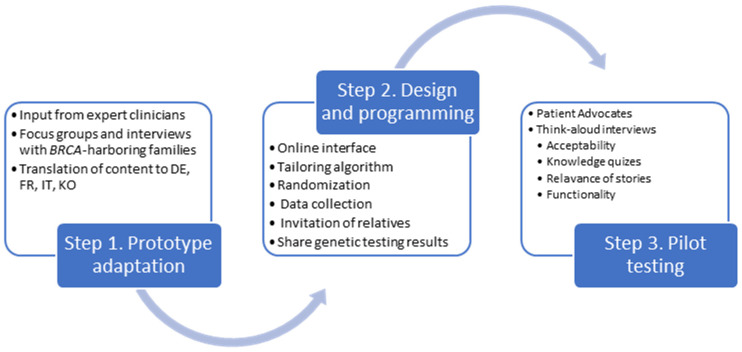
The steps of adaptation and pilot testing of the Family Gene Toolkit.

The adaptation of the Family Gene Toolkit prototype followed a three-step process (Figure 1). In step 1, we updated and adapted the prototype based on newer evidence regarding cancer risks associated with *BRCA1*/*BRCA2* pathogenic variants and feedback from expert clinicians, researchers, and individuals from *BRCA1/BRAC2*-harboring families. In step 2, we designed and programmed the functions of the web application that will host the Family Gene Toolkit. In step 3, we tested the acceptability and usability of the new Family Gene Toolkit. The study protocol has been approved by appropriate Ethics Committees (BASEC 2016-02052 and SEVIRB 2020-0833-006) and is publicly available (NCT04214210; KCT0005643).

### 2.1. Step 1. Adaptation of the Prototype

The cultural adaptation of the Family Gene Toolkit involved collecting narrative data through focus groups and in-depth interviews. This process included individuals from families harboring *BRCA1* or *BRCA2* pathogenic variants and took into account Swiss and Korean legislation, health insurance policies, and cultural values. Narratives evolved around cancer risk and genetic testing, risk management, and family communication [8], and informed culturally appropriate message framing, identified tailoring elements, and illustrative stories. The adapted content was translated from English to German, French, Italian, and Korean, following the established methods for the translation of health-related messages [43].

The adapted Family Gene Toolkit was reviewed by clinicians involved in genetic counseling in Switzerland and Korea and experts in health communication, nursing, psychology, and sociology. Clinicians and experts were identified through the Schweizerischen Arbeitsgemeinschaft für Klinische Krebsforschung (SAKK) Network for Cancer Predisposition Testing and Counseling, through the Oncoplastic Breast Consortium [44], and through the CASCADE (NCT03124212) [45] and the K-CASCADE (KCT0005643) [46] consortia. Experts met in small groups and evaluated the alignment of the content with the current guidelines regarding the management of *BRCA1* and *BRCA2*-associated cancer risks, the consistency of the translated medical and genetic terms with terminology used in clinical practice, the appropriateness and relevance of messages and illustrative stories, and the appearance, organization, and clarity of the slideshow.

### 2.2. Design and Programming

The interface of the adapted Family Gene Toolkit was based on design principles for navigability and user experience of web applications [47,48,49]. To design the main content of the Family Gene Toolkit, we used a readily available e-learning product (www.articulate.com accessed on 5 September 2023) that offers software solutions to create an interface accessible from a computer, tablet, and smartphone. This was integrated with a customized system to manage user accounts, provide localization into various languages, allow users to invite relatives to the system, and track user activities for research purposes.

### 2.3. Acceptability and Usability Testing

The final version of the Family Gene Toolkit underwent acceptability (favorable attitude toward and satisfaction with the intervention) and usability testing (testing for functional errors) through an iterative process. Acceptability and usability testing was conducted with patient advocates in each country and new members from families harboring *BRCA1*/*BRCA2* pathogenic variants recruited from the CASCADE and K-CASCADE consortia. Acceptability was tested using “think-aloud” interviews, an established method for participants to voice their thoughts, feelings, and opinions while they are completing each task of the web application [50]. During the “think-aloud” interviews, participants provided verbal feedback on various aspects of the Family Gene Toolkit. This included assessing its usefulness and the reading level and comprehension of messages from the lay public, evaluating the effectiveness of visual illustrations and narratives in conveying key concepts, and offering suggestions for improving the context, layout, pictures, and color scheme. “Think-aloud” interviews were conducted in five languages (German, French, Italian, Korean, and English). 

Usability testing is an established technique aiming to systematically test the navigability of a tool prior to its distribution [51,52]. Usability testing assessed two main aspects. First, the ability of participants to use all functions and features of the web application. Second, the ease and user-friendliness of navigation across various devices, including laptops, tablets, and smartphones. This evaluation included opening the platform, navigating through each module, and interacting with its components. Participant feedback was elicited either in person or in virtual sessions via Zoom. Sessions were recorded, and team members took notes for each step. Feedback from each cycle informed the modifications that were tested in the subsequent cycle.

The acceptability and usability of the Family Gene Toolkit were also assessed with a 14-item Likert scale (1 = low to 7 = high). After completing the “think-aloud” protocol, participants were asked to rate their overall satisfaction with the application. Satisfaction included aspects such as the helpfulness and clarity of the content, expressing whether they desired additional information in specific content areas, evaluating the user-friendliness of navigation, and sharing their thoughts on the format and appearance of the slideshow. We used descriptive statistics, such as medians and interquartile range (IQR), to describe participants’ demographic characteristics and summarize the acceptability and usability data. All computations were performed in R software, version 3.6.3 [53]. Narrative data from the “think aloud” interviews were analyzed using content analysis [54] from two members of the research team in each country.

## 3. Results

### 3.1. Adaptation of the Prototype

Insights for culturally sensitive message framing, tailoring, and illustrative stories were gained from 68 women (46 Swiss and 22 Korean) harboring *BRCA1*/*BRCA2* pathogenic variants who provided narrative data. Most women in both countries were well-educated, married or in a relationship, and had at least one previous cancer diagnosis. The only difference was that Swiss women were more likely to be employed outside the household (Table 1). Participants emphasized the significance of including certain elements in the web-based platform. For example, they highlighted the importance of information about cancer risk for both sexes and suggested a comprehensive explanation of the genetic counseling and testing process that would address common concerns that people might have. They also stressed the importance of incorporating information about prophylactic surgeries, such as mastectomy and salpingo-oophorectomy, into the platform, as these details are often overlooked in genetic counseling sessions. In addition, the inclusion of testimonials and personal stories would greatly enhance the platform by creating a sense of community and providing reassurance to users.

Feedback was also elicited from 31 clinicians and experts (24 Swiss and 7 Korean) representing different linguistic regions (*n* = 11 German-, *n* = 8 French-, *n* = 5 Italian-, and *n* = 7 Korean-speaking). Feedback was elicited in two rounds of 4 mini focus groups (a total of 8 focus groups) in Switzerland and 7 individual interviews in Korea. Teams in each country met independently and together to finalize the culturally sensitive adaptation of the content, message-framing, and illustrative stories. This iterative process took place from January 2022 to April 2023. The adapted content was first developed in English at an eighth-grade reading level and was translated into German, French, Italian, and Korean. Clinicians and researchers provided feedback at least twice during the adaptation process, both for the English and translated versions.

**Figure 2:**
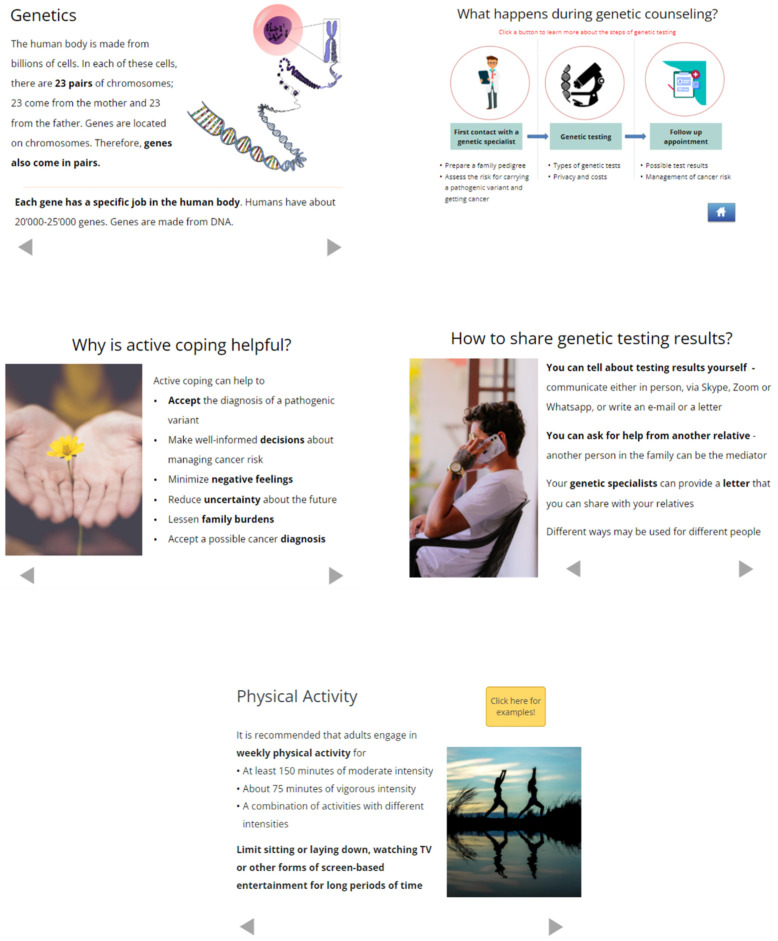
Examples from the five modules of the Family Gene Toolkit.

The adapted Family Gene Toolkit included the original four modules and a newly developed fifth module addressing cancer risk management. The modules and the interface were supplemented with multiple interaction options to enhance user engagement, i.e., quizzes and assessments, illustrative stories, and resources to connect with psychologists, family therapists, nutritionists, and specialists for smoking cessation. Pictures were carefully selected for each country to enhance the displayed messages and increase relatedness based on age, sex, and race (Figure 2 and Supplementary Materials). The content was adapted as follows:

*Genetics and cancer:* This module provides basic information about the risk of developing HBOC-associated cancers with and without the contribution of *BRCA1* or *BRCA2* pathogenic variants and the modes of inheritance of these variants. The content was updated to emphasize the association of *BRCA1*/*BRCA2* variants with prostate, pancreatic, and possibly other types of cancer [4]. A link to available genetic services and a quiz were added to increase user interaction.

*Genetic counseling and testing:* This module is intended only for relatives who did not have genetic testing. It describes the genetic counseling process and provides updated information regarding panel and targeted testing, country-specific laws for the protection of genetic information and associated costs, and illustrative stories about the advantages and disadvantages of genetic testing. It enables interactive pedigree visualization and includes a knowledge quiz, a link to available genetic services, and a list of questions relevant to the pre- and post-testing consultations.

*Coping with cancer risk*: This module explains the difference between active coping and avoidance and focuses on the importance of active coping and family support in HBOC. Testimonials from individuals with *BRCA1* or *BRCA2* pathogenic variants are used to demonstrate active coping with lifelong personal and family challenges associated with HBOC. The module was updated with links to genetic services in each country, while information on accessing psychological services and support groups increase user interaction.

*Family communication:* This module is intended only for individuals with *BRCA1* or *BRCA2* pathogenic variants. It explains the legal framework regarding the family-mediated communication of test results in each country, describes common issues that arise during this process, and provides practical tips to avoid family conflicts. Communication skills are enhanced with a prescriptive approach to the disclosure of testing results. The module was updated with culturally sensitive testimonials from individuals with *BRCA1* or *BRCA2* pathogenic variants. Links to the available genetic and psychological services in each country increase user interaction.

*Cancer risk management:* This module offers generic information on how testing results can inform prevention and screening for cancers known to be associated with *BRCA1*/*BRCA2*-associated HBOC. It also provides information exclusively for women, i.e., risk-reducing surgeries, breast reconstruction, esthetic flat closure, and risks and benefits of anti-hormonal treatment and oral contraceptives. The content includes country-specific information on a balanced diet, recommended levels of physical activity and alcohol consumption, and encourages smoking cessation. A quiz and links to available nutritional and smoking cessation services increase user interaction.

### 3.2. Design and Programming

The Information Technology (IT) Services team from the Department of Clinical Research at the University Hospital of Basel, Switzerland, developed a custom web application to facilitate the following processes:

- Enable secure, password-protected log-in for potential participants, assess eligibility, and provide a web-based consent form;
- Deliver a baseline questionnaire to collect information used for message tailoring and for evaluating outcomes;
- Facilitate the invitation of at-risk relatives to the web application via email and SMS messaging;
- Randomize participants either to the Family Gene Toolkit or a comparator website. At-risk relatives will be automatically assigned to the same group as the person who invited them to the study;
- Deliver the Family Gene Toolkit or the comparator, a non-interactive generic website that provides basic information related to HBOC;
- Deliver an evaluation questionnaire to assess satisfaction with the content of the Family Gene Toolkit and the comparator and with the technical aspects of navigating the web application;
- Deliver a follow-up questionnaire that will be used for evaluating primary and secondary outcomes related to family communication of testing results and cascade testing of relatives.

The function of the web application that facilitates cascade testing of at-risk relatives is the ability to send SMS and email messages to at-risk relatives and links to available genetic and other healthcare services. The web application will track the number of invitations sent to relatives, the proportion of at-risk relatives who receive an invitation over the number of relatives potentially eligible for cascade testing, and the number of invitations that have been accepted by invited relatives. The web application will track access and use various indicators of “intervention dose”, e.g., time spent on each session and engagement with interactive content. Instructions are provided on the main menu page, and users are directed through the content with “next “, “previous”, and “home” buttons. All collected data will be securely stored and routinely backed up in protected servers of the University Hospital, Basel, Switzerland.

### 3.3. Acceptability and Usability Testing

Acceptability and usability testing of the adapted Family Gene Toolkit was tested with 18 women (13 Swiss and 5 Korean) who participated in the “think-aloud” interviews. The sample included mostly well-educated women who were employed outside their households. There was one untested relative, while the remaining 17 women had genetic testing and were identified as carriers of a *BRCA1* or *BRCA2* pathogenic variant (Table 2).

Participants in the ‘think aloud’ interviews in both countries engaged with the entire content of the Family Gene Toolkit and provided favorable feedback for the navigation. They clicked at least once on the links with the list of available genetic specialists while navigating each module. They also clicked on the links with the list of psychological and nutritional services and patient support groups. Most participants provided positive feedback for the testimonials in their respective language that illustrated active coping and the challenges of communicating testing results. They referred to the module for family communication as ‘fresh and helpful’. Participants also appreciated the engagement with quizzes and found the comprehensive explanation of the correct answer useful. The newly developed module on cancer risk management was highly appreciated, especially the information about the various types of breast reconstruction after mastectomy, which was referred to as ‘empowering’. Almost all participants rated the content as highly acceptable (Table 3). They perceived that the length of each module and the amount of information was well-balanced and that the information was useful and easy to understand and made them think of ways to help their family.

Usability testing showed that navigating through the entire content of the Family Gene Toolkit took, on average, 55 min (range: 25–110). Completing the baseline questionnaire took approximately 15 min, and the evaluation questionnaire took approximately 3 min. Most participants (78%) stated that they would have liked to see the Family Gene Toolkit before or at the time they had genetic testing. An area for further improvement expressed by about 33% of participants included the possibility of a personalized risk assessment for various cancers rather than a range of risks. Table 4 presents illustrative quotes that convey satisfaction with the web application and suggestions for improvement.

## 4. Discussion

This study presents the adaptation of the Family Gene Toolkit and results from acceptability and usability testing with members from Swiss and Korean families harboring *BRCA1* or *BRCA2* pathogenic variants. An essential component of the adaptation process was the engagement and collaboration of multiple stakeholders, i.e., clinicians, content experts, patient advocates, and members of families harboring *BRCA1*/*BRCA2* pathogenic variants from Switzerland and Korea. The two teams worked together to create tailored and culturally sensitive messages and an interactive, user-engaging interface.

The adapted Family Gene Toolkit will be delivered via a website in an asynchronous communication pattern. While real-time interaction between family members and clinicians may be lacking, along with the chance for immediate feedback, asynchronous communication offers maximum flexibility and can support implementation upscaling. It allows tailoring the delivery time to the circumstances and preferences of individuals with pathogenic variants and at-risk relatives and the possibility of reviewing the content multiple times. Another advantage is the ability to reach a wider audience across all time zones. Given the linguistic and cultural diversity of Swiss-based families (62% German, 23% French, 8% Italian, 1% Romansh, and 23% of other ethnic and racial origin) [55], it is expected that the Family Gene Toolkit will be accessed by many families in German, French, Italian, and English-speaking countries. Korean-speaking families worldwide may also benefit from the intervention since the Korean diaspora accounts for more than 14% of the Korean population [56].

The tailoring algorithm is based on genetic testing status, with different content delivered to carriers of *BRCA1*/*BRCA2* pathogenic variants and untested relatives. The Family Gene Toolkit can be accessed outside of a clinical setting as an additional product to assist initial and follow-up discussions with genetic specialists during the continuum of genetic care. Individuals with pathogenic variants can review information about cancer genetics, which may have been overwhelming during genetic consultation [8]. They can also review the steps for effective communication and use the communication guide to create a tailored algorithm for disseminating testing results to at-risk relatives. Although the web application does not necessarily foster interaction among family members, the prescriptive approach increases awareness about maintaining healthy boundaries in family communication, which can promote positive family dynamics [57].

Similarly, untested relatives are introduced to complex information. They become aware of the possible risks and advantages of genetic testing, and they can also compile a list of questions before consulting a specialist. This proactive approach aids in addressing misunderstandings and encourages well-informed decision-making. The web application can help relatives process this information without the perceived pressures of a clinical setting. Information about available genetic specialists is expected to increase self-efficacy and remove barriers related to accessibility of services [58,59].

All participants receive information about active coping strategies. These strategies are linked to various positive outcomes, such as enhanced mental well-being, increased feelings of empowerment and control, and greater resilience when dealing with challenges [60,61]. The Family Gene Toolkit is also designed to enhance participants’ self-reflections on how their own values and practices impact their families and social environment. Reflexivity about one’s practices is crucial for promoting the capacity to make choices according to one’s values in the context of one’s intimate family and social life [62,63]. All participants are provided with information concerning lifestyle adjustments and cancer risk management. This includes details about medication, risk-reducing surgeries, early detection through screening, and options for breast reconstruction. This newly developed module is among the few interventions designed to address the informational needs of individuals with *BRCA1*/*BRCA2* pathogenic variants and their untested relatives across the continuum of care [9].

Accessibility and usability testing showed that the adapted Family Gene Toolkit is a well-designed, well-functioning, and scalable tool. All users indicated that the web application provided useful information they wished they had when first confronted with their genetic testing results and increased susceptibility to cancer. The use of testimonials helped participants relate to the content based on their life trajectory, medical history, and family dynamics. One possible improvement is the ability to provide individualized predictions for various cancer risks rather than a range of risks. Another possible improvement is to integrate large language models (such as Generative Pretrained Transformers) into the Family Gene Toolkit to guide the tailoring algorithm and increase the usability of the web-based platform through natural language processing [64].

One limitation is that the current version of the Family Gene Toolkit is limited to individuals with *BRCA1*/*BRCA2* pathogenic variants and does not cover other genes associated with HBOC. While our sample size was sufficient for acceptability and usability testing, further testing in a randomized trial with a parallel control group (RCT) and a larger sample will provide evidence of its efficacy in increasing rates of cascade testing among at-risk relatives. The RCT will also inform deep message tailoring, for example, whether participants choose to view some content based on their own coping style. It is also envisioned that data collected from the RCT will help determine a further need to add narration. At this stage, the team decided against this option because integrating speech technologies using the web speech API is time-consuming and costly due to continuous updates and limited browser support [65]. Moreover, privacy considerations must be considered if APIs send data from medically-focused websites to central servers for translation. Another limitation is that most participants were well-educated, implying that they were at least moderately skilled in using a web application. This may have contributed to positive usability ratings. The sample also included exclusively females since no males expressed willingness to test the web application. The RCT will also provide insights on how to engage males with HBOC-associated genetic testing and reduce the gender-based disparity for this syndrome [66].

## 5. Conclusions

Given the constantly changing landscape of cancer genomics and the lack of genetic specialists, there is a clear need for digital tools designed to support the communication of genetic testing results and facilitate cascade testing of at-risk relatives. Web applications can significantly contribute to ease and convenient access to health-related information, supporting the genetic counseling process and patient satisfaction in the continuum of genetic care [26,67]. In Switzerland, only 25% of patients with breast cancer and a strong family history have received genetic counseling for HBOC-associated variants [68]. In Korea, genetic counseling is not yet mandated, although it is offered in many tertiary hospitals. The Family Gene Toolkit can provide valuable assistance to families in order to cope with and manage their cancer risk, communicate effectively about pathogenic variants, and increase rates of cascade testing among at-risk relatives.

## Figures and Tables

**Table 1:** Characteristics of the 68 women who provided narrative data for culturally sensitive content adaptation and message framing.

Characteristic	Swiss Sample *N =* 46	Korean Sample *N =* 22
Age (mean, range)	50 (32–72)	42 (27–68)
Linguistic region	(*n*, %)	(*n*, %)
French-speaking	25 (54%)	Not applicable
German-speaking	14 (31%)	
Italian-speaking	7 (15%)	
Education		
Compulsory/High school/Technical school	28 (61%)	7 (32%)
University/Post-graduate degree	18 (39%)	15 (68%)
Employment		
Yes	36 (78%)	8 (36%)
No	10 (22%)	14 (64%)
Marital status		
Married/Partnered	35 (76%)	15 (68%)
Divorced/Separated/Widowed	7 (15%)	1 (5%)
Single	4 (9%)	6 (27%)
Previous cancer diagnosis		
Yes (breast, ovarian, other)	29 (63%)	17 (77%)
No	17 (37%)	5 (23%)

**Table 2:** Characteristics of the 18 women who participated in the “think-aloud” interviews for acceptability and usability.

Characteristic	*N* = 18
Age (mean, range)	51 (28–70)
Linguistic region	
French-speaking	7 (39%)
German-speaking	5 (28%)
Italian-speaking	1 (6%)
Korean-speaking	5 (28%)
Education	
Compulsory/High school/Technical school	10 (56%)
University/Post-graduate degree	8 (44%)
Employment	
Yes	12 (67%)
No	6 (33%)
Marital status	
Married/Partnered	13 (72%)
Divorced/Separated/Widowed	2 (12%)
Single	3 (16%)
Previous cancer diagnosis	
Yes (breast, ovarian, other)	12 (67%)
No	6 (33%)

**Table 3:** Acceptability of the Family Gene Toolkit.

Question	Median (IQR) *
The Family Gene Toolkit had helpful information for…	
risk factors for hereditary breast and ovarian cancer syndrome	7 (1)
the genetic counseling and genetic testing process	7 (1)
how to find genetic services	7 (1)
cancer screening for people at higher risk	7 (1)
tips for family communication of genetic testing results	7 (0)
tips for family support in genetic cancer syndromes	7 (0)
The Family Gene Toolkit…	
was easy to understand	7 (1)
took too much time to review	3 (4)
made me nervous	1 (1)
was important to me	7 (1)
made me think about ways to help my family	6 (2)
was not useful to me	1 (1)
I would suggest this study to other people	7 (1)
The study was important	7 (1)

Note: * Likert point scale (1: Strongly Disagree; 2: Disagree; 3: Somewhat Disagree; 4: Neutral; 5: Somewhat Agree; 6: Agree; 7: Strongly Agree).

**Table 4:** Quotes demonstrating overall satisfaction with the Family Gene Toolkit.

Topic	Question	Quotes from “Think Aloud” Interviews
Content	How did you like the content of the Family Gene Toolkit?	*“I’d like to show it to my son…there is a lot of information about men.”*
		*“I had no idea that there are medications that could reduce cancer risk.”*
		*“I found the quiz really helpful; it helps the information to stick in my mind.”*
Missing information	Is there any information that you needed but it was not addressed?	*“I would like to find more information about my personal cancer risk. And a specific risk estimate.…That would be more helpful for me.“*
Timing of intervention	When do you think is the best time to deliver this information?	*“I wish I had this intervention before I even started thinking about genetic testing and dealing with my cancer risk.”*
		*“I think this intervention would be more helpful when someone is just being diagnosed with the mutation.”*
Navigation	How easy or difficult was it to navigate the web application?	*“I expected that clicking on the arrow would take me back to the main menu, but it didn’t. It was not clear to me what this ‘home’ button was.”*
		*“The quizzes are very nice, but I would also like to have a detailed explanation when I selected the correct answer.”* (This comment was addressed in subsequent interviews.)
		*“It was not clear that I could find more links and see more stories when I clicked on words that were blue and bold.”*
Overall satisfaction	Overall, what do you think about the information covered in the Family Gene Toolkit?	*“The intervention is very well-done, with clear and comprehensive information, and made me feel that I want to read more.”*
		*“It contains everything and exhausted all the information.”*
		*“Overall, I think the intervention is nice, has beautiful pictures, and is user-friendly. I had no trouble navigating through and finding what I needed.”*
	Overall, was there something that you did not like?	*“The intervention was very informative and well-structured, but I feel that this is very long.”*
		*“I think it would be stressful for some people to get this information. Maybe the intervention needs some more positive content.”*
		*“I felt burdoned to tell my relatives. To me, it was hard to share results with my family members.”*

Italics present excerpts from narrative data demonstrating participants’ perceptions about the Family Gene Toolkit.

## Data Availability

The CASCADE and K-CASCADE Consortia are open to collaborations with national and international researchers. We invite interested parties to contact the study team through website (https://swisscascade.ch/en/contact-2/, accessed on 5 September 2023) to discuss project ideas, data access, and the submission of research concepts to the Scientific Board. Templates for data requests and further information on the study are available (https://swisscascade.ch/en/research-projectdata-request/, accessed on 5 September 2023).”

## Supplementary Materials

The following supporting information can be downloaded at: https://www.mdpi.com/article/10.3390/cancers15184485/s1.
