# Supplementary material for: Acceptability and Usability of the Family Gene Toolkit for Swiss and Korean Families Harboring BRCA1/BRAC2 Pathogenic Variants: A Web-Based Platform for Cascade Genetic Testing
Source: Cancers (Basel). 2023 Sep 9;15(18):4485. doi: 10.3390/cancers15184485 (PMC10527353; doi:10.3390/cancers15184485)
Supplement: Supplementary file 1 [file cancers-15-04485-s001.zip › cancers-2577248-supplementary.pdf]

Examples of the interface and context of the web-based platform

I. German version

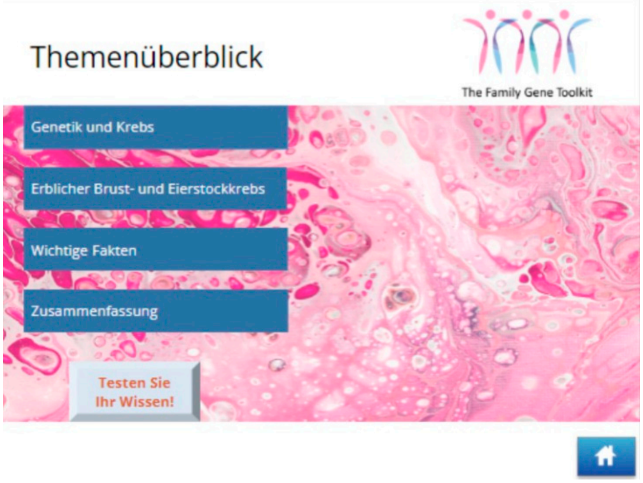

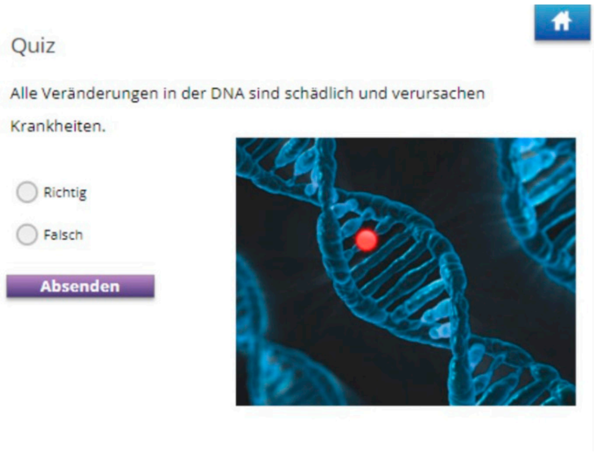

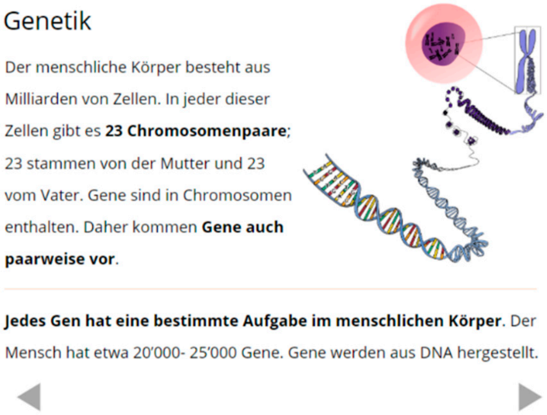

II. French version

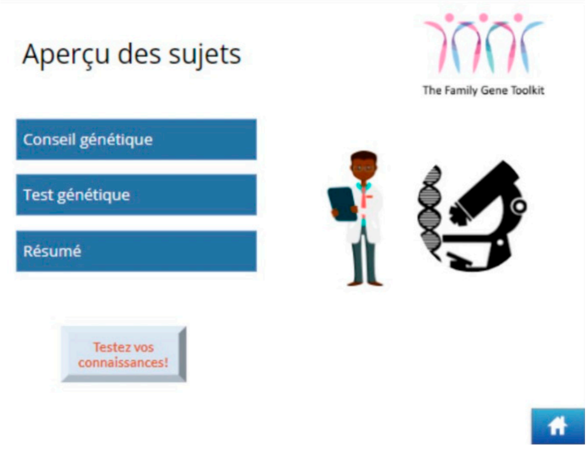

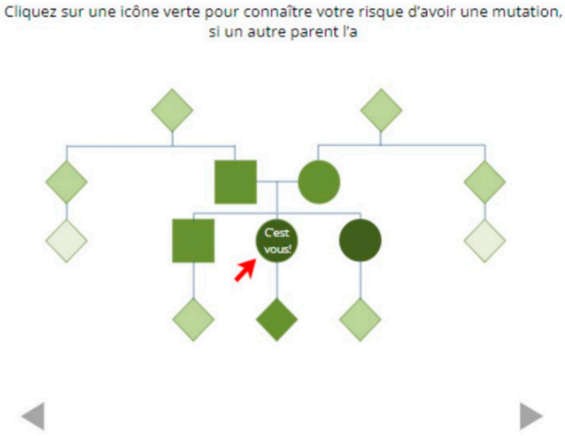

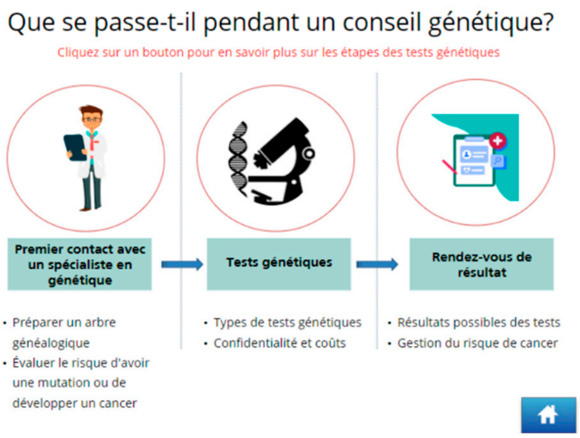

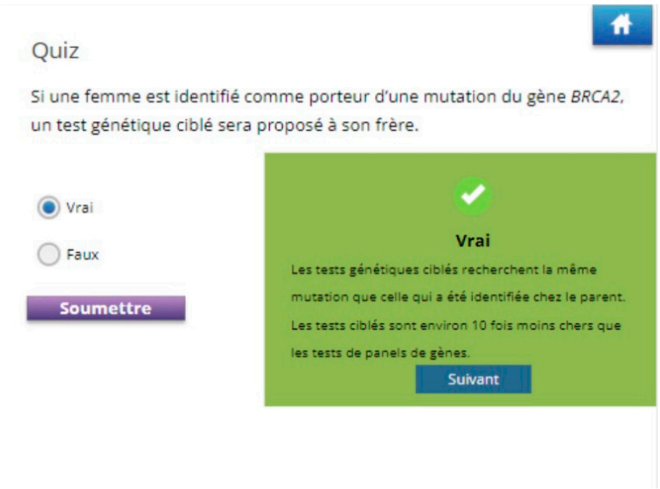

III. Italian version

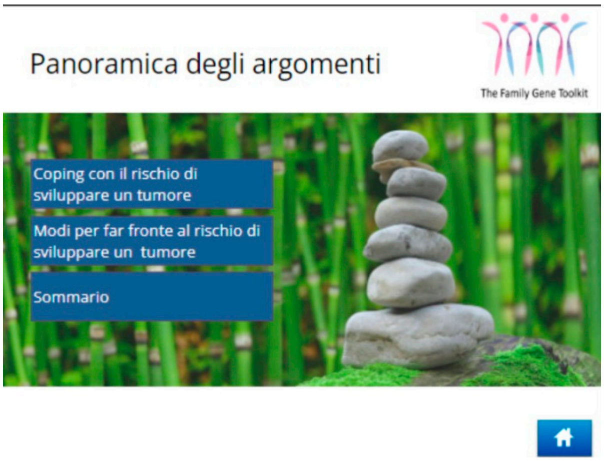

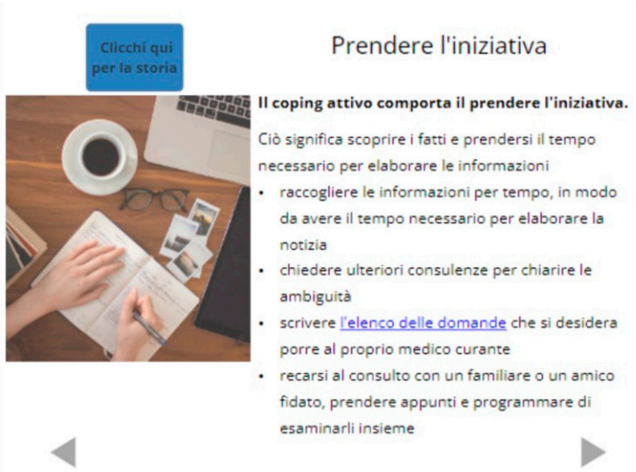

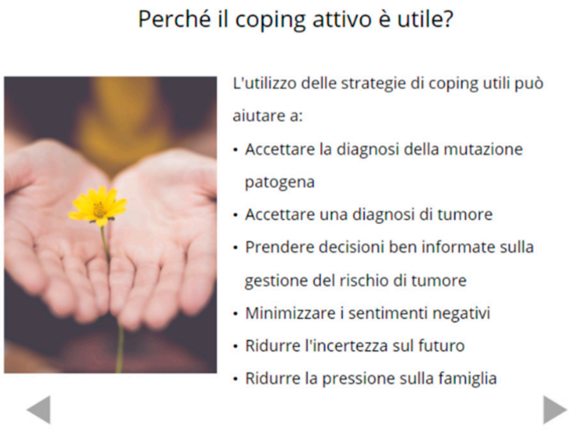

IV. English version

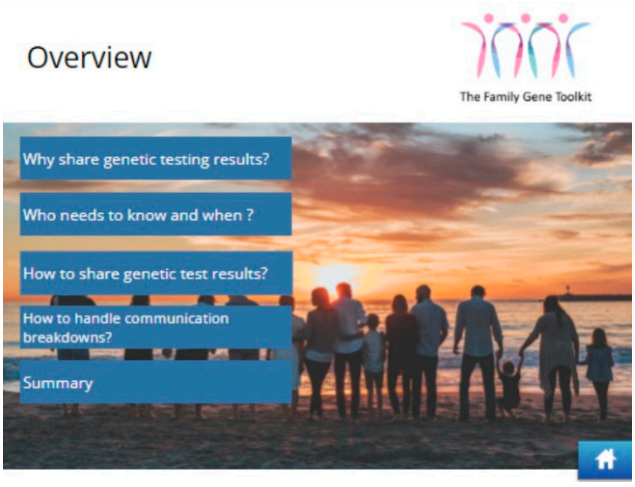

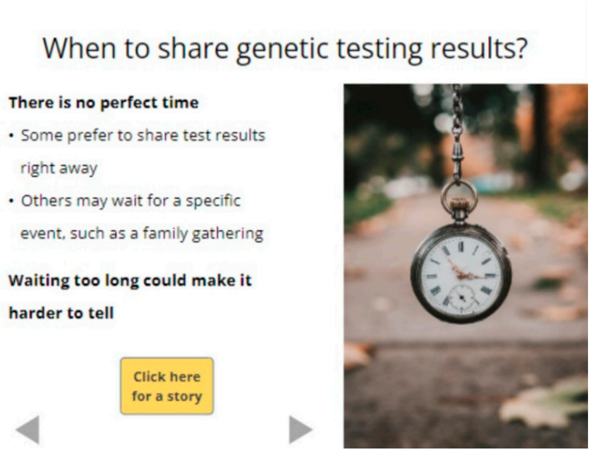

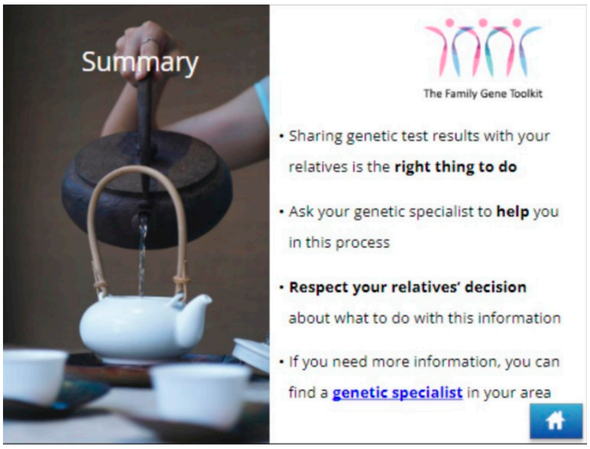

V. Korean version

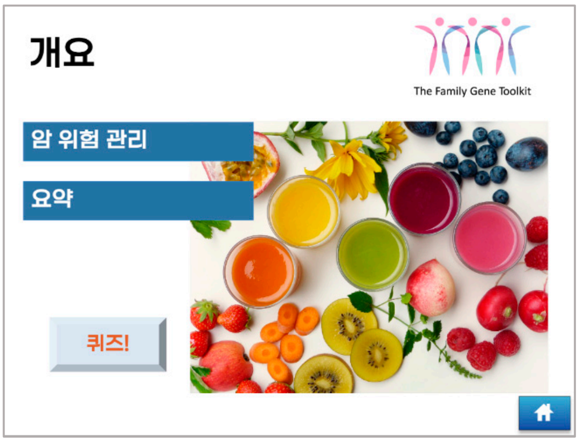

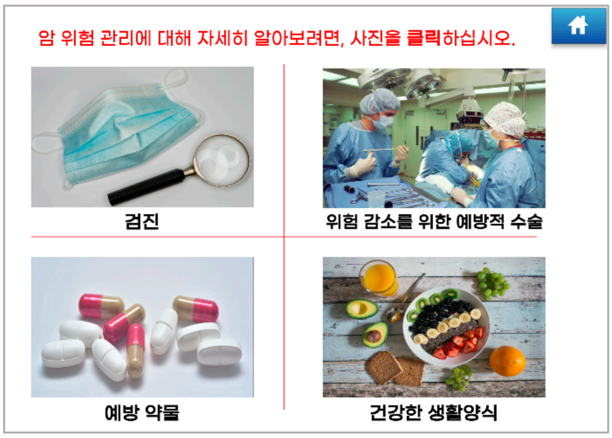

퀴즈

선별검사(암 조기 검진)를 받는 것과 위험감소를 위한 치료를 받는 것은 *BRCA1* 또는 *BRCA2* 병인성 변이가 있는 모든 남성과 여성에게 적용된[illegible]

- 참
- 거짓

제출하기

정답

선별검사를 받는 것과 위험 감소를 위한 치료를 받는 것은 모든 남성과 여성에게 적용되는 것은 아닙니다. 선별검사는 특정 병인성 변이, 성별, 그리고 다른 건강 상태에 따라 달라집니다. 또한 개인적인 상황, 선호도, 가치관에 따라 달라질 수 있습니다. 유전학 전문가는 다른 선택지를 제시할 수도 있고, 개인별 가장 좋은 선택지가 무엇인지 결정하도록 도울 수 있습니다.

계속하기

요약

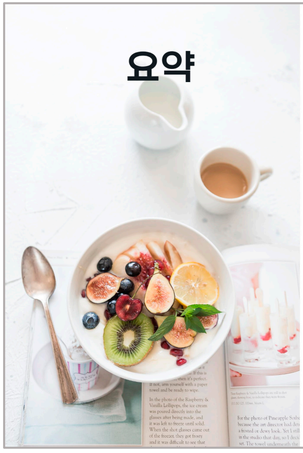

The Family Gene Toolkit

의학적 지침(조기 발견, 위험 감소 예방적 수술, 약물 복용)과 건강한 생활 양식의 **조합**은 궁극적으로 **암 위험을 줄일 수 있습니다.**

신체활동

예를 보려면, 여기를 **클릭** 하십시오.

성인은 **주1회 신체활동(운동)**에 참여할 것을 권장합니다.

- *BRCA* 보인자에서 적당한 운동은 유방암의 위험 감소
- 일주일에 적어도 3번 이상 , 하루에 30분 이상 땀이 날 정도의 운동은 유방암 위험 감소

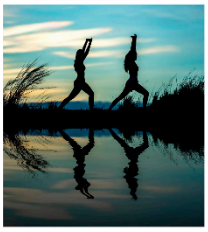

장시간 앉아 있거나 누워 있는 것, TV 또는 스마트폰 등 화면 기반 엔터테인먼트를 보는 것을 제한합니다.
